# Differential Alloreactivity: Lessons Learned From a Singular HLA Locus

**DOI:** 10.1111/tan.70489

**Published:** 2025-12-09

**Authors:** Esteban Arrieta‐Bolaños

**Affiliations:** ^1^ Institute for Experimental Cellular Therapy, University Hospital Essen Essen Germany

**Keywords:** antigen presentation, haematopoietic cell transplantation, HLA‐DP permissive mismatches, immunobiology, immunogenetics, immunopeptidome, major histocompatibility complex, peptide‐binding motifs, T‐cell epitopes, T‐cell receptors

## Abstract

Alloreactivity entails the recognition of cells and tissues from one individual as foreign by T cells and other immune effectors from another individual. Alloreactive immune responses play an important role in various clinical contexts, in particular in transplantation. Major drivers of these responses are the highly immunogenic, non‐self HLA molecules. However, the immunogenicity of these allogeneic HLA molecules has been observed to vary according to certain immunobiological and immunogenetic parameters, leading to the concept of differential alloreactivity. Recent progress in unveiling the underpinnings of this phenomenon has been made for the frequently mismatched HLA‐DP allotypes, whose singular genomic, structural and population genetics characteristics offer an ideal scenario for these investigations. Studies in the HLA‐DP context have highlighted the immunopeptidome overlap between self and non‐self HLA allotypes, as well as its editing by non‐classical class II chaperones HLA‐DM and HLA‐DO, as a main determinant of their immunogenicity likely via indirect effects of thymic education. Recent evidence suggests that these observations could also be extended to alloresponses directed against HLA molecules encoded by other loci. How these functional characteristics of HLA molecules shape allorecognition by T‐cell subsets, and how they translate into different clinical consequences in the context of transplantation will be the subject of the present review.

**Abbreviations**
3′UTR: 3′ untranslated region
ARD: antigen recognition domain
CDR3: complementarity‐determining region 3
CIITA: class II transactivator
CLIP: class II invariant chain–derived peptide
DEAV/GGPM: motifs (positions 84–87) in the HLA‐DP peptide‐binding groove
HCT: haematopoietic cell transplantation
HIV: human immunodeficiency virus
mHAg: minor histocompatibility antigen
MHC: major histocompatibility complex
PBM: peptide‐binding motif
TCR: T‐cell receptor

## 1 Introduction

Alloreactivity, that is, the process of recognition of cells and tissues from one individual as foreign by immune cells (or their effector molecules, e.g., antibodies) of another individual, plays an important role in various clinical contexts, including pregnancy, blood component transfusion and transplantation of cells and tissues. In transplantation, the main mediators of alloreactive responses are recipient and donor‐derived T cells, which respond to antigens on the graft or recipient tissues. These antigens can be in the form of polymorphic peptides presented by matched major histocompatibility complex (MHC) molecules, in humans also called HLA, which constitute the so‐called minor histocompatibility antigens (mHAg). In addition, T cells have an exquisite capacity to directly recognise the highly immunogenic non‐self HLA molecules presenting a large number of allopeptides. In fact, it has been estimated that up to 10% of circulating T cells can elicit direct alloreactive responses against non‐self HLA [1, 2], a frequency that is 10^2^ to 10^3^‐fold higher than the precursor frequency of T cells specific for any single foreign‐peptide–self‐HLA complex. Traditionally, T‐cell alloreactivity had been thought to be independent of thymic education based on self HLA. However, the immunogenicity of allogeneic HLA molecules has been observed to vary according to certain immunobiological and immunogenetic parameters that influence the alloreactive precursor frequency and the strength of T‐cell alloresponses, leading to the concept of differential alloreactivity.

Recent progress in unveiling the underpinnings of this phenomenon has been made for the class II HLA‐DP allotypes [3], whose singular genomic, structural and population genetics characteristics offer an ideal scenario for these investigations. Studies in the HLA‐DP context have highlighted the immunopeptidome overlap between self and non‐self HLA allotypes, as well as its editing by non‐classical class II chaperones HLA‐DM and HLA‐DO, as a main determinant of their immunogenicity. Recent evidence suggests that these observations could also be extended to alloresponses directed against HLA molecules encoded by other loci. The present review will explore how these functional characteristics of HLA molecules shape the strength and diversity of allorecognition by T‐cell subsets, and how these translate into different clinical consequences in the context of transplantation.

## 2 HLA‐DP: A Singular Locus

The HLA‐DP molecules constitute one of the three major HLA class II proteins together with HLA‐DR and HLA‐DQ. Similar to other class II molecules, HLA‐DP allotypes are constituted by heterodimers formed by alpha and beta chains encoded by separate loci. The two chains form a peptide‐binding groove in which the presence of four main pockets with the ability to accommodate specific amino acid residues determines the peptides that will preferentially bind to each allotype. These molecules play a central role in adaptive responses by presenting peptide antigens to CD4+ T cells, and are expressed mainly by immune antigen‐presenting cells under steady‐state conditions. However, other tissues can acquire expression of these molecules upon stimulation from extrinsic factors including pro‐inflammatory cytokines like interferon‐γ. As for other class II genes, HLA‐DP expression is closely regulated by the MHC class II transactivator CIITA. While the presentation of exogenous peptides is a cardinal feature of HLA class II molecules, most peptides presented by them actually derive from the degradation of intracellular proteins, even in antigen‐presenting cells [4]. The peptides presented by HLA‐DP and other classical class II molecules are edited by non‐classical HLA‐DM molecules. In certain cell types and states [5, 6], another non‐classical class II molecule, HLA‐DO, acts as an inhibitor of HLA‐DM, thereby influencing the presentation of peptides by the class II molecules [7, 8]. Even though it was one of the last major HLA molecules to be described, the role of HLA‐DP as a target for alloreactive T‐cell responses and a classical transplantation antigen was promptly recognised [9, 10, 11, 12, 13].

Despite its similarities to other class II molecules, HLA‐DP possesses a series of particularities that set it apart, including characteristics at the genomic, structural, functional and population genetics levels. On a genomic level, the HLA‐DPA1 and HLA‐DPB1 loci lie centromeric to the other major HLA loci in the short arm of chromosome 6, and, due to the presence of at least three major recombination hotspots between these and HLA‐DQB1 [14, 15, 16, 17], there is weaker linkage disequilibrium between HLA‐DP and other HLA loci. This results in less linkage of variants in otherwise conserved extended HLA haplotypes. In terms of genetic diversity, HLA‐DPB1 has fewer described alleles (*N* = 2911) and proteins (*N* = 1670) compared to other class II beta chain loci, although these numbers have recently increased and are now approaching those of the HLA‐DQB1 locus (IMGT/HLA database version 3.62, 2025‐10) [18]. Sequence analyses have suggested that variants at HLA‐DPB1 have arisen through recombination and point mutation of a few core alleles to produce the allele repertoire seen today [19]. At the structural level, motif shuffling across five hypervariable regions in the HLA‐DPB1 gene forms the basis of variation within HLA‐DP allotypes [20], which show more intra‐locus similarity than other HLA molecules. In line with this, HLA‐DPB1 proteins have lower amino acid variation compared to other class II beta chains [19], resulting in lower HLA evolutionary divergence in patient cohorts [21, 22, 23]. On a population level, alleles at HLA‐DPB1 are highly skewed to a handful of variants making up most of the variation observed in most human groups. The number of observed second‐field alleles is low across geographic regions compared to other major HLA loci except HLA‐DQB1 [24]. Interestingly, while for most HLA loci allele frequency data provide strong evidence for balancing selection at the allele level, allele frequencies at HLA‐DPB1 show a pattern compatible with neutral evolution (by genetic drift) or directional selection in most human populations [25]. Despite this, evidence for balancing selection has been detected at the nucleotide and amino acid sequence level, in particular on HLA‐DP serologic categories [26].

These peculiarities have been instrumental in the advances made in understanding basic principles of alloreactivity as explained in detail in the sections below. In particular, the presence of high‐frequency alleles covering most of the allelic diversity in the population, the reduced level of structural diversity involving the reshuffling of conserved motifs at HLA‐DPB1 hypervariable regions, and the high prevalence of HLA‐DPB1 mismatches in the clinical context of haematopoietic cell transplantation (HCT) [27] due to the low linkage disequilibrium with other major loci have fuelled studies into the determinants of alloreactive T‐cell responses against HLA‐DP allotypes and facilitated experimental approaches to study this phenomenon directly.

## 3 Differential Alloreactivity: A Role for the Immunopeptidome

The extent to which peptides being presented by mismatched HLA molecules participate in the direct alloresponses against them has been a matter of debate. While allorecognition had been postulated by some to differ from conventional recognition of peptide‐HLA complexes, leading to models suggesting that the direct binding of the T‐cell receptor (TCR) to the allogeneic MHC would be enough to elicit T‐cell activation [28], several findings have supported similarities between allo‐ and conventional recognition, giving the peptides a central role in this process [29, 30, 31]. As a matter of fact, the peptide‐dependency of many alloreactive TCRs has been well documented [32]. Perhaps the most striking illustration of the importance of the HLA peptide repertoires in immune responses came from a context in which no allogeneic cells or tissues were involved. In the context of hypersensitivity reactions against the anti‐retroviral drug abacavir, elegant work demonstrated that these exaggerated immune responses were due to the lodging of the drug in the HLA‐B*57:01 antigen‐recognition domain (ARD) causing a shifting in the peptide‐binding characteristics of the groove [33, 34, 35, 36, 37]. The altered peptide repertoires presented to autologous CD8+ T cells in vivo induce a strong response against self that is analogous to a direct alloreactive response. In fact, further work demonstrated that the peptide repertoire deregulation by abacavir exposure could induce de novo HLA‐B*57:01 allorecognition by HIV‐specific T cells [38].

In alloresponses against HLA‐DP, early observations on the cross‐reactivity of HLA‐DP9‐specific T‐cell clones recognising different HLA‐DP allotypes [39] led to the postulation of so‐called T‐cell epitopes underlying those patterns [40]. In an attempt to better understand the nature of these epitopes, alloresponses against the HLA‐DP9 molecule were studied in in vitro models involving mutations at specific positions in its ARD [41]. When the consequences of these amino acid changes on allorecognition patterns were assessed, it became clear that both the specific position as well as the nature of the amino acid change affected the allorecognition of the HLA‐DP9 molecule by CD4+ T‐cells. Some of these changes led to an effective abrogation of the alloreactive T‐cell activation. By modelling the interaction between the specific position in the HLA‐DP ARD, the TCR and the bound peptide, it became clear that most of the induced mutations found to have a significant impact on the allorecognition would affect the interaction between the HLA molecule and the peptides being presented, as had been previously suggested [42, 43, 44]. Hence, this work also supported a peptide‐centric allorecognition of HLA‐DP explaining the alloreactivity patterns observed in the T‐cell clones investigated.

As mentioned above, a key observation in alloreactivity against HLA‐DP molecules was that the magnitude of in vitro T‐cell responses was variable depending on the allotype being recognised [45]. This differential immunogenicity correlated with allele groups based on an ARD amino acid‐based functional distance between them [41]. Consequently, the question that followed was whether the peptides being presented could explain the differences in the strength of the alloreactive responses observed. To investigate this, experimental data on the repertoires of peptides presented by different HLA‐DP allotypes, that is, their immunopeptidomes [4], were generated in order to examine their correlation with the strength of alloresponses against these molecules [46]. In these experiments, HLA‐DP molecules naturally expressed on the cell surface of B‐lymphoblastoid cell lines, as well as on HeLa cells transduced to express single allotypes, were subjected to immunoprecipitation, followed by acid elution and sequencing of the bound peptides by liquid chromatography and tandem mass spectrometry [46, 47]. The repertoires consisting of several thousand peptides were then compared to investigate their similarity. These analyses demonstrated that the immunopeptidomes of some allotypes showed a significant overlap, while this was negligible between others. Interestingly, these significant overlaps were present in allotype pairs that were known to elicit weaker alloreactive responses in in vitro experiments [45, 46, 48]. This observation suggested a role for the immunopeptidome overlap in determining the frequency of alloreactive precursors against a non‐self HLA molecule. In this model, structurally and functionally close allotypes would share a substantial number of peptides presented in a similar way, leading to the deletion of many T cells capable of recognising them during thymic negative selection. This indirect effect of thymic selection on alloreactivity, that is, effective deletion of clones with alloreactive capacity during negative selection on self‐HLA, would then become negligible if the repertoires of presented peptides are largely divergent [3]. These findings were also in line with previous reports implicating endogenous self‐peptides in shaping the alloresponse by T cells [30, 31, 49].

To further illustrate the central role of the immunopeptidomes in the differential alloreactive responses against HLA‐DP allotypes, similar experiments were carried out comparing the repertoires of peptides presented in the presence of the non‐classical class II molecule HLA‐DM with those retrieved from cells lacking it [46]. HLA‐DM helps remove the class II invariant chain‐derived peptide (CLIP) and functions as a class II peptide editor, effectively influencing which peptides are bound and presented by HLA‐DP [7]. When HLA‐DM editing is active, the presentation of high‐affinity (i.e., HLA‐DM‐resistant) peptides is favoured, while low‐affinity (i.e., HLA‐DM‐sensitive) peptides are removed. Importantly, the absence of HLA‐DM editing did not affect the levels of expression of HLA‐DP allotypes, nor did it result in significant levels of CLIP peptide retention. Even though more CLIP peptides were bound by HLA‐DP allotypes in HLA‐DM‐negative cells, CLIP accounted for less than 10% of the total peptide pools [46]. This is in contrast to HLA‐DR, where the absence of HLA‐DM leads to changes in the bound peptide motif and preferential CLIP binding [50].

**FIGURE 1:**
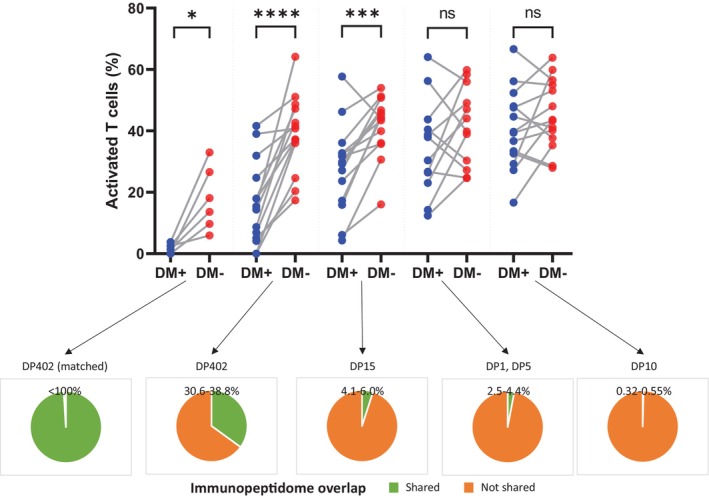
The effects of HLA‐DM editing on differential alloreactivity depend on the degree of immunopeptidome overlap. Alloresponses from CD4+ T cells from healthy donors against different HLA‐DP allotypes were measured using an in vitro model with HLA‐DP‐transduced HeLa cells as stimulators as previously described [46, 48, 52, 53]. Responders were typed as self‐HLA‐DPB1*04:01 homozygous, DPB1*02:01, 04:01, or DPB1*04:01, 04:02, and were stimulated with HeLa cells expressing allotypes HLA‐DP1, DP402, DP5, DP10, or DP15, in the presence and absence of HLA‐DM. The magnitude of the in vitro alloreactive response is depicted as the percentage of activated CD4+ T cells upon restimulation in CD137 upregulation assays. Blue dots depict alloresponses against the allotypes in the presence of HLA‐DM (DM+), whereas paired responses against the same allotype in the absence of HLA‐DM (DM−) are shown in red. The level of immunopeptidome overlap between the self‐HLA‐DP of the T‐cell responders and the stimulating allotype expressed by the HeLa cells in the in vitro assays was calculated as the proportion (%) of peptides that are shared between the allotypes from the responders and the target allotype with respect to the combined immunopeptidome of the responder allotypes (green). The overlap in alloresponses against mHAg presented by matched HLA‐DP402 is set to < 100%. Immunopeptidome data was derived from HLA‐DP‐bound peptides directly retrieved from K562 cell lines transduced with the relevant HLA‐DP allotypes (data publicly available in the PRIDE partner repository, dataset identifier PXD030591) [47]. The absence of HLA‐DM regulation of the immunopeptidome has a strong impact on the magnitude of the alloreactive response against HLA‐DP allotypes with higher overlaps, while this impact diminishes when the immunopeptidome divergence is large. ns, not significant. *p < 0.05, ***p < 0.001, ****p < 0.0001.

In line with this, when comparing the immunopeptidome of the same HLA‐DP allotype expressed in the same genetic background of HeLa cells in the presence and absence of HLA‐DM, the results showed only a partial overlap of the peptide repertoires, with significant expansions in their number and changes in the intracellular compartment origin of the retrieved peptides when HLA‐DM was absent [46]. While peptides bound by HLA‐DP allotypes in the presence of HLA‐DM stemmed mostly from vesicular, exosomal and granular compartments, those presented in its absence were predominantly of cytoplasmic, organelle and membrane‐derived protein origin. This is in accordance with the observation that presentation of endogenous peptides is also a common feature for class II molecules [4]. Strikingly, these immunopeptidomic changes led to an abrogation of the differential alloreactivity observed for structurally related HLA‐DP allotypes, effectively increasing the immunogenicity of these allotypes to the levels observed for more divergent variants [46]. Both the lack of enrichment of conserved CLIP peptides in the immunopeptidomes of HLA‐DP, which for HLA‐DR has been associated with reduced immunogenicity of leukaemic cells [51], as well as the broadening of the potentially antigenic peptide pool likely underlie the expansion of the T‐cell alloresponses against structurally related HLA‐DP allotypes. Importantly, these observations were made not only in samples from healthy, unsensitised individuals, but also confirmed with donor‐derived CD4+ T cells isolated from patients 1 year after HLA‐DP‐mismatched HCT [46]. Interestingly, the impact of HLA‐DM on the magnitude of alloreactive responses is indeed inversely correlated with the degree of immunopeptidome divergence between HLA‐DP allotypes (Figure 1). These results suggested that the differential alloreactivity against HLA‐DP molecules depends not only on the degree of immunopeptidome overlap between self and non‐self allotypes, but also on the presentation of an HLA‐DM‐edited repertoire of peptides. This observation also suggests that the indirect effects of thymic education on the peripheral frequencies of T cells alloreactive to non‐self HLA‐DP occur predominantly through negative selection in the presence of HLA‐DM. Similarly, while its natural antagonist HLA‐DO [54] has been implicated in thymic selection in animal models [55], these data would also suggest a reduced role for HLA‐DO in the shaping of the alloresponses against HLA‐DP. Indeed, the concomitant presence of HLA‐DM and HLA‐DO reproduced the significant increase in alloresponses against otherwise low immunogenic HLA‐DP allotypes [46]. Nevertheless, whether HLA‐DO plays a more subtle yet appreciable role in modulating the alloreactive capacity of peripheral T‐cell repertoires deserves more investigation. Overall, these observations established the degree of HLA‐DM‐edited immunopeptidome overlap between self and allogenic HLA‐DP as a main factor influencing the alloresponse against these molecules.

## 4 Immunopeptidome Divergence: Consequences for Alloreactive T Cells

**FIGURE 2:**
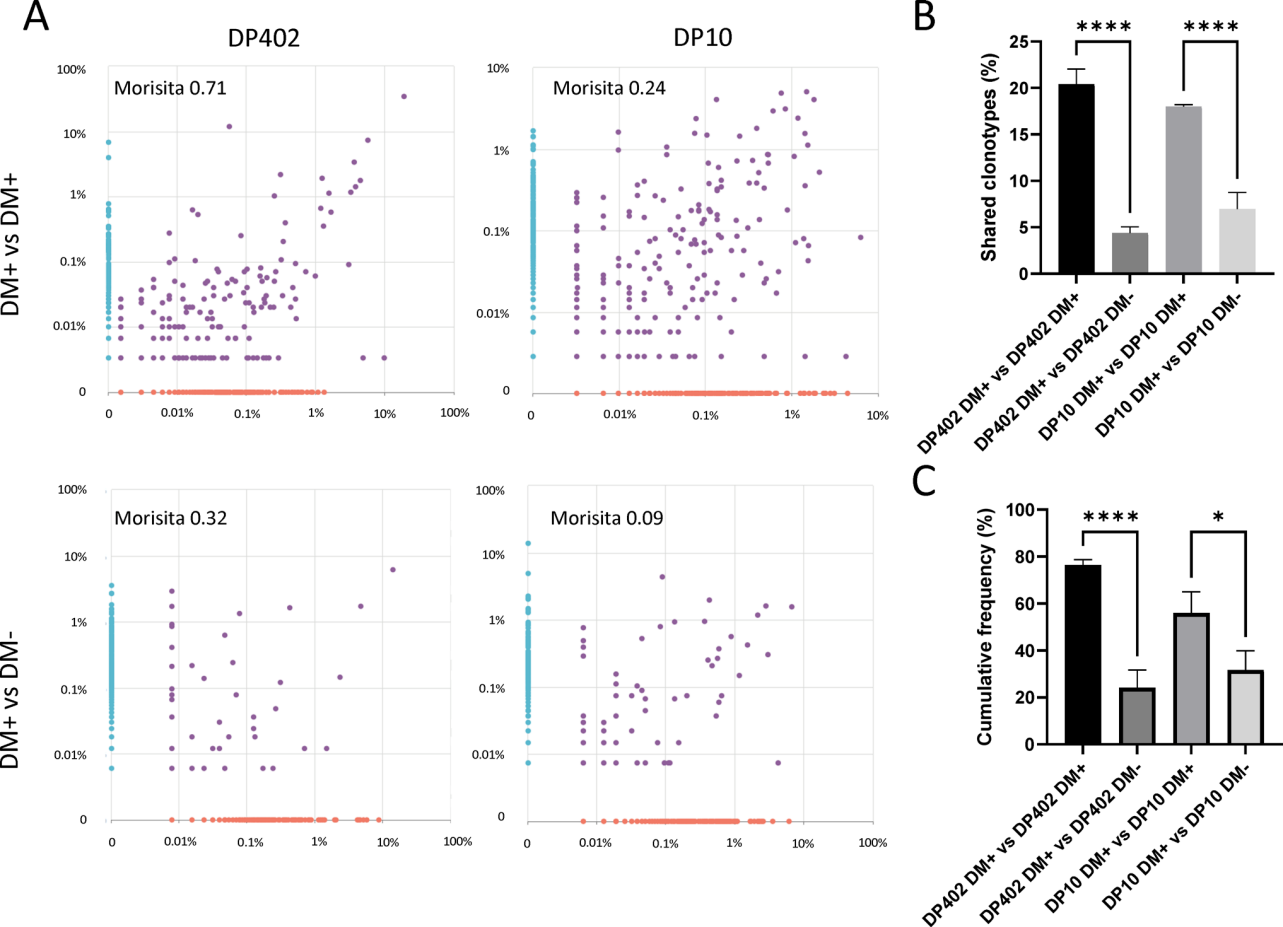
HLA‐DM‐dependence of anti‐HLA‐DP alloreactive TCR repertoires. TCR next‐generation sequencing of in vitro‐raised alloreactive CD4+ T cells responding against an allotype with low immunopeptidome divergence, that is, HLA‐DP402, or against highly divergent HLA‐DP10 in the presence (DM+) and absence (DM−) of HLA‐DM editing was applied to parallel cultures from the same responder (self HLA‐DP401 homozygous) [46]. (A) While parallel cultures against the same allotype in the presence of HLA‐DM (top row) lead to a significant number of clonotypes observed at similar frequencies in both cultures (purple dots) and a hence a strong repertoire similarity as measured by the Morisita index [56], comparison of the TCR repertoires enriched in cultures against the same allotype but in the presence and absence of HLA‐DM (bottom row) results in fewer overlapping clonotypes and a significant reduction in repertoire similarity. Both the percentage of shared clonotypes (B) and their cumulative frequency (C) are significantly reduced when the repertoires of parallel cultures in the presence of HLA‐DM are compared to those of cultures with and without HLA‐DM for both allotypes. *p < 0.05, ****p < 0.0001.

In addition to the effects on the overall magnitude of the alloresponse, the degree of immunopeptidome overlap and its editing by HLA‐DM also influenced qualitative aspects of these immune responses against HLA‐DP molecules. First, congruent with more extensive deletion of alloreactive CD4+ T cells, the TCR diversity in alloresponses against HLA‐DP allotypes with high immunopeptidome overlap was significantly lower when compared to alloresponses against allotypes with higher immunopeptidome divergence [46]. Second, the diversity of TCRs responding against an allotype with high immunopeptidome overlap was significantly increased when HLA‐DM editing was absent to levels that were comparable to those observed against highly divergent allotypes [46]. This correlates with the fact that the expansion of the peptide repertoires and their diversification would reduce the indirect effects of thymic education by a structurally related allotype on the alloreactive T‐cell repertoire, leading to stimulation of more clones. Moreover, further analysis of the clonotypes involved in these responses shows that, while parallel cultures from the same responder against the same HLA‐DP allotype in the presence of HLA‐DM lead to significant overlap of the repertoire of in vitro‐expanded alloreactive CD4+ T cells regardless of the immunopeptidome divergence between self and non‐self, the absence of HLA‐DM significantly reduces this overlap (Figure 2). Interestingly, T cells expanded against HLA‐DP in the absence of HLA‐DM were not efficiently stimulated by the same allotype in the presence of HLA‐DM, whereas the opposite was true for those raised against the allotype in the presence of HLA‐DM [46]. This demonstrates that the deregulation of the class II immunopeptidome leads to the asymmetrical recruitment of different sets of alloreactive T cells responding against the same HLA‐DP allotype, highlighting again the central role of the immunopeptidome and its regulation in shaping also qualitative aspects of the alloresponse.

Newer analyses into the effects of immunopeptidome overlap on alloreactive T cells provide further insights into the mechanisms involved in alloreactivity against HLA‐DP. The relative role of naïve vs. memory T cells in alloreactive responses has been a matter of debate [57, 58, 59, 60]. However, the alloreactive cross‐reactivity of pathogen‐specific memory T cells is well documented, both within and across HLA loci [61, 62]. We have recently investigated the role of CD4+ T cell subsets in alloresponses against HLA‐DP allotypes with different degrees of immunopeptidome divergence. By separating memory and naïve T cells from healthy individuals, we have been able to demonstrate that, while naïve cells predominate in alloresponses against mHAg presented by matched‐HLA‐DP and against HLA‐DP allotypes with high immunopeptidome overlap with self, memory CD4+ cells are effectively recruited into the alloresponse against mismatched HLA‐DP allotypes with high immunopeptidome divergence [63]. Interestingly, the deregulation of the immunopeptidome presented by matched‐HLA in the absence of HLA‐DM also led to increased participation of memory cells in alloresponses against mHAg. These results suggest that the expansion of the peptide repertoire not observed during thymic education increases the probabilities of pathogen‐specific memory CD4+ T cells recognising allogeneic HLA molecules and mHAg in the context of matched HLA‐DP. The degree of immunopeptidome divergence would hence determine the magnitude of the participation of memory T cells in alloresponses from unsensitised individuals.

Another relevant observation supporting the important consequences of immunopeptidome divergence in alloreactive responses against HLA‐DP relates to the capacity of stimulating the same T cell clones. As explained above, alloreactive CD4+ T cells expanded in vivo [39] or raised in vitro [64] show predictable cross‐reactivity patterns with other HLA‐DP allotypes [65, 66]. These patterns were later correlated with differential alloreactivity against these molecules [45] and the degree of immunopeptidome overlap between them [46, 47, 66]. More recent investigations have further studied the nature of the cross‐reactivity between HLA‐DP allotypes. In previously unsensitised individuals, most circulating alloreactive T cells have been shown to be of very low frequency and found within the deep peripheral repertoire [2]. In vitro alloreactive responses from different individuals against the same HLA‐DP allotype do not result in the enrichment of similar T‐cell clonotypes, and the overlap between alloresponses from the same individual against structurally divergent allotypes is very low [52]. Conversely, our investigations with CD4+ T cells isolated from the same individual have shown that in vitro stimulation with different non‐self allotypes that have a significant sharing of peptides between them [66] can indeed lead to the enrichment of alloreactive T cells expressing the same CDR3 sequences. These shared TCR sequences are often found among the top‐10 clonotypes in both post‐culture repertoires (manuscript in preparation). The fact that the same alloreactive clonotypes can be enriched independently from among millions of circulating T cells in non‐primed naïve individuals constitutes strong evidence that immunopeptidome overlap between two HLA allotypes is a central factor influencing the recruitment of specific alloreactive repertoires.

## 5 Differential Alloreactivity: Implications Beyond HLA‐DP

The findings presented above have been instrumental in our understanding of the biological factors determining the role of alloreactivity against HLA‐DP in clinical contexts such as HCT [3]. Here, in vivo responses against mismatched HLA‐DP allotypes have long been known to influence the outcome of these therapies [67, 68, 69, 70, 71]. Currently, both the tolerability of these incompatibilities, as well as their influence on the curative efficacy of HCT can be understood in terms of the degree of immunopeptidome divergence between patient and donor [27, 41, 46, 47, 48, 72, 73, 74, 75]. Allotypes with high immunopeptidome overlap are generally better tolerated, reducing the risk of complications such as graft‐versus‐host disease [48]. Conversely, allotypes with higher immunopeptidome divergence can lead to more effective graft‐versus‐malignancy effects, positively impacting the therapeutic effects of transplantation [72]. Hence, the degree of immunopeptidome divergence at HLA‐DP has been proposed as an actionable factor that could be intelligently harnessed in the management of patients with haematological diseases treated with HCT [27, 76, 77, 78].

The insights derived from these investigations into the immunobiology of HLA‐DP mismatches have been further developed and applied to alloreactivity against other HLA loci. In the case of HLA class I, while incompatibilities are avoided whenever possible, mismatched transplants are performed in patients lacking well‐matched donors. In fact, such mismatched donor transplantations are becoming much more common in the current HCT era [79]. In vitro investigations have shown that class I allotypes matched for their ARD induce limited alloreactive responses [80, 81, 82] and represent tolerable mismatches in HCT [83]. Different from HLA‐DP, increased allelic diversity and a lack of immunopeptidomic data obtained in the same genetic background limit the applicability of immunopeptidome overlaps in the assessment of alloreactivity against class I allotypes. To circumvent these limitations, their peptide‐binding motifs (PBM), that is, the amino acid signatures of the peptide repertoires bound by the HLA molecule, have been proposed as proxies for their immunopeptidome [84, 85]. Based on the hypothesis that PBM similarity would correlate with lower immunopeptidome divergence, PBM groups based on hierarchical clustering have been devised to classify alleles and predict their immunogenicity according to the genetic makeup of the responder. Significant improvements in both the quality and the quantity of immunopeptidomic data for a wide range of allotypes from all major HLA genes in recent years [84, 86] have allowed for an expansion of the applicability of PBM‐based mismatch immunogenicity prediction. This approach has been applied as a novel way of assessing the tolerability of HLA mismatches in clinical HCT, showing an important role for PBM matching in patient survival after mismatched HCT [85, 87]. Experimental work is underway to determine whether PBM grouping of HLA class I allotypes also correlates with in vitro alloreactivity. These findings with PBM mismatches reinforce the notion that immunopeptidome divergence plays a central role also in HLA class I mismatch differential immunogenicity and tolerability, as described above in the context of HLA‐DPB1. Similar approaches involving the use of PBM to assess the immunogenicity of the other class II loci are being developed [88], setting the stage for a pan‐HLA immunopeptidome divergence‐based understanding of direct alloreactivity.

## 6 Perspectives and Outlook

The investigations presented above support a central role for the degree of immunopeptidome overlap between HLA allotypes in direct alloreactive responses. While these principles seem to be generalisable across the HLA genes, a number of different factors could nevertheless affect their correlation with differential alloreactivity. First, while the immunopeptidome is largely defined by the amino acid conformation in the ARD of the HLA molecule, the actual repertoire of peptides being presented at any time might be affected by environmental conditions such as the type of cell and the tissue it is part of, the cellular microenvironment and transcriptional or proteomic status, changes in the antigen‐processing machinery or external influences like inflammation. This suggests that the degree of immunopeptidome divergence might be a dynamic variable rather than a steady‐state condition, which could hence affect differential alloreactivity. Future research into how alloreactive responses are modified by these immunopeptidomic factors is warranted.

Similarly, since the class II immunopeptidomes are highly influenced by the presence of HLA‐DM, and, in some contexts, the balance between HLA‐DM and HLA‐DO [54], any factors influencing the editing of the immunopeptidome by these chaperones are likely to affect alloreactive responses. HLA‐DM is known to be polymorphic, and some variants have been demonstrated to display differences in their peptide‐editing activity, resulting in differential activation of CD4+ T cells [89, 90]. Although their frequency in different human populations is low, recent work has shown that the number of these variants is higher than previously thought [91]. We are currently investigating how this natural variation in HLA‐DM activity affects differential alloreactivity against HLA‐DP allotypes. In addition to the natural variation in HLA‐DM, there is accumulating evidence that allotypes of the same HLA class II molecule can have intrinsic differences in their sensitivity to HLA‐DM‐mediated peptide editing and/or in their affinity or binding to CLIP peptides [92, 93, 94, 95, 96]. Since immunopeptidome editing by HLA‐DM appears to be a central feature in differential alloreactivity against class II, both differences in editing activity across HLA‐DM variants and intrinsic variability in allotype susceptibility to HLA‐DM could affect their immunogenicity. More research is needed in order to better understand this interplay and its clinical consequences.

Another factor that could modulate the intensity of alloreactive responses pertains to differences in the levels of expression of HLA allotypes [97]. For HLA‐DP, investigations in hepatitis B suggested a role for the expression levels of different allotypes defined by the presence of a polymorphism in the HLA‐DPB1 3′ untranslated region (UTR; rs9277534 G/A) in immune responses [98]. This role was later investigated in HCT and shown to be associated with increased risk of graft‐versus‐host disease [99, 100]. While the interaction between expression levels and immunopeptidome‐based differential alloreactivity is difficult to disentangle because of the strong linkage between the 3′ UTR rs9277534 G/A dimorphism [53, 101] and the 84–87 DEAV/GGPM motifs in the HLA‐DP peptide‐binding groove P1 pocket, it is conceivable that higher or lower levels of expression might affect the immunogenicity of HLA molecules in alloreactive contexts. More research to determine the interaction between expression levels and immunopeptidome divergence is hence warranted.

Although PBMs constitute an efficient way to approximate the overall immunopeptidome similarity between HLA allotypes with implications for HCT outcome [85, 87, 88], studies in highly related class I allotypes have demonstrated that HLA ‘micropolymorphism’ (i.e., sequence differences of just a few amino acids) can influence not only the repertoire of displayed peptides, but also their quantity, as well as the stability and conformation of the peptide‐HLA complex [102]. These immunopeptidomic differences, not necessarily evident from PBM data, have the capacity to drive clinically relevant alloreactive T‐cell responses [103]. How much these finer immunopeptidome differences influence differential alloreactivity within and across PBM groups, and which of these translate into clinically relevant consequences warrants further scrutiny. Overall, the significant improvements in mass spectrometry sensitivity and the availability of high‐quality immunopeptidome data for all major HLA loci seen in recent years [84, 86] are likely to continue in the foreseeable future. These improvements will eventually result in the extension of immunopeptidome data to all globally common HLA allotypes, and potentially allow for a cell type‐ or tissue‐specific immunopeptidome overlap‐based analysis. Continuous review of HLA allotype affinities and their role in alloresponses in view of these novel data is hence warranted.

Finally, the knowledge related to the role of HLA‐DM in alloresponses against HLA‐DP has opened new avenues for translational approaches aimed at harnessing alloreactivity to tackle leukaemia relapse after HCT. While co‐expressed with classical HLA class II molecules in most tissues [104], it is well described that leukaemic cells can naturally lose HLA‐DM expression [105] and that lack of CLIP removal from class II molecules, a surrogate for HLA‐DM activity, correlates with clinical outcome [106, 107, 108]. Moreover, haematological malignancies derived from the B‐lymphoid lineage [109, 110], as well as some myeloid neoplasms [106], express the natural antagonist of HLA‐DM, HLA‐DO. Such diseases would be natural candidates for targeted, HLA‐DM‐sensitive alloreactive TCR‐based therapies. In general, there is emerging interest in the role of the immunopeptidome in cancer and translational approaches to harness it in order to improve targeted cellular and immunotherapies for malignant diseases [111].

## 7 Conclusion

**FIGURE 3:**
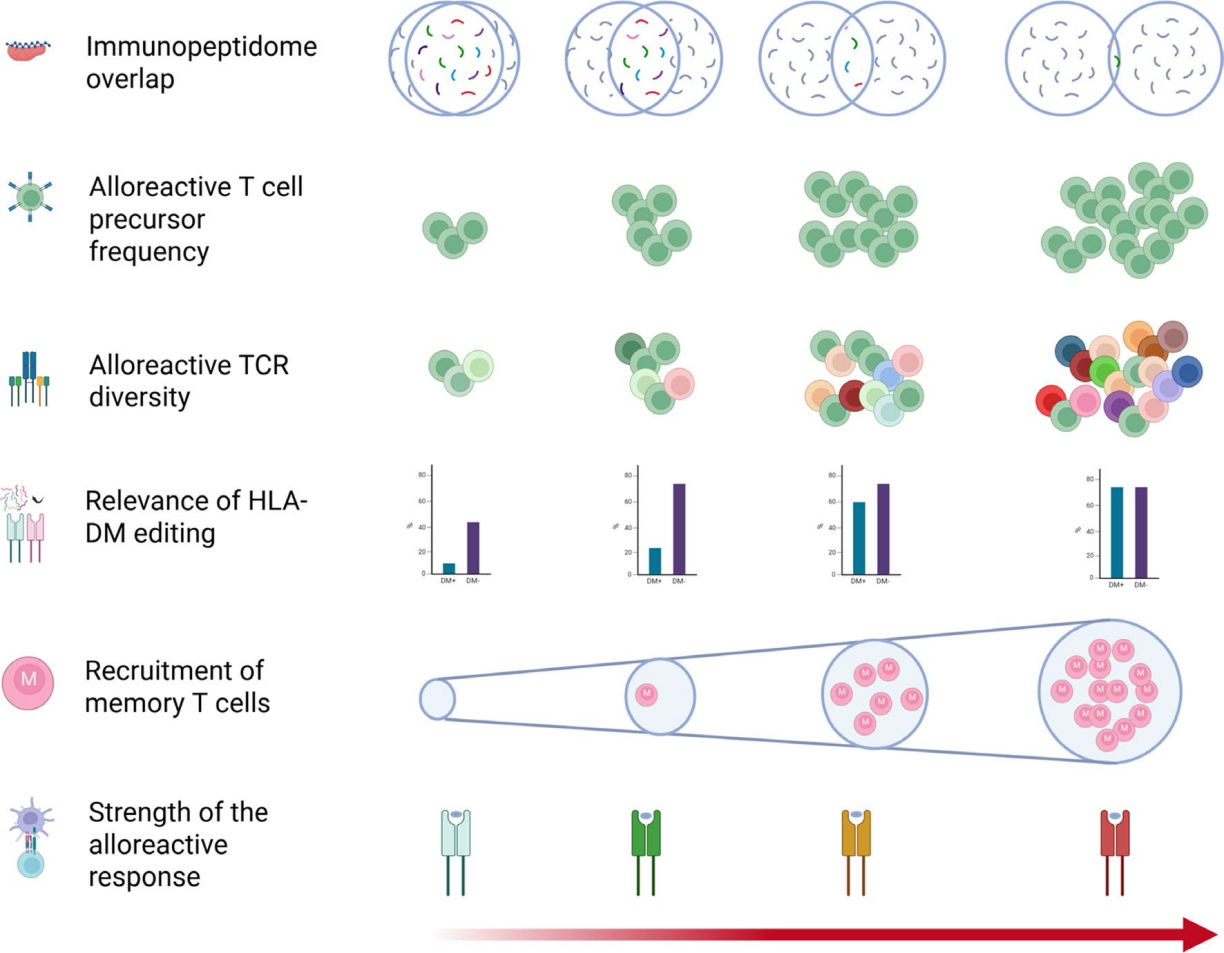
Biological correlates of differential alloreactivity against HLA‐DP. Alloreactive responses against HLA‐DP allotypes vary in strength according to the functional properties of their peptide‐binding grooves. Functionally close allotypes have a significant overlap of their immunopeptidomes. This overlap results in lower frequency and TCR diversity of alloreactive CD4+ T‐cell precursors. Conversely, functionally distant allotypes have lower or almost negligible immunopeptidome overlaps, which are able to stimulate more and more diverse alloreactive T cells. Weaker alloresponses against functionally close allotypes depend on the peptide‐editing activity of HLA‐DM; when HLA‐DM is absent, or its activity is reduced either intrinsically or by HLA‐DO, the alloresponse against these allotypes is significantly increased. For functionally distant allotypes, the presence or absence of HLA‐DM plays a lesser role in the overall magnitude of the alloresponse (see Figure 1), although it does change the TCR repertoire responding against the allotype (see Figure 2). Finally, while memory cells have a limited participation against matched‐HLA‐DP‐restricted mHAg and functionally close allotypes, they are efficiently recruited to the alloresponse against functionally divergent HLA‐DP.

The investigations into the differential immunogenicity of HLA‐DP allotypes have contributed significant knowledge, both basic as well as clinical, to our understanding of the immunobiology of alloreactivity and its consequences in clinical contexts. Through a combination of in vitro experiments and high‐throughput technologies providing direct insights into of the determinants of HLA allotype immunogenicity, these works have highlighted the role of the immunopeptidome overlap in differential alloreactivity against HLA molecules. Moreover, the critical role of the class II immunopeptidome editing by the chaperone HLA‐DM, further reinforces the relevance of the peptide repertoires in defining both quantitative, as well as qualitative aspects of alloreactive T‐cell repertoires responding against an HLA class II allotype (Figure 3). The realisation that the immunopeptidome presented by HLA‐DP allotypes is at the basis of the phenomenon of differential alloreactivity and mismatch tolerability has opened the possibility to extrapolate these principles to other major HLA loci. Finally, approaches building on this knowledge and aimed at manipulating the immunopeptidome to harness alloreactivity constitute the next frontier for translational immunogenetic research in cellular and immunotherapies.

## Author Contributions

E.A.‐B. summarised the literature and wrote the manuscript.

## Conflicts of Interest

The author declares no conflicts of interest.

## Data Availability

The data that support the findings of this study are available on request from the corresponding author. The data are not publicly available due to privacy or ethical restrictions.
